# Concomitant Rupture of the Tendons of Extensor Pollicis Longus and Extensor Indicis Proprius Following Volar Plating for a Distal Radius Fracture

**DOI:** 10.7759/cureus.76596

**Published:** 2024-12-29

**Authors:** Shimul Dey, Matthew Venus

**Affiliations:** 1 Plastic and Reconstructive Surgery, University Hospitals Coventry and Warwickshire National Health Service (NHS) Trust, Coventry, GBR; 2 Plastic Surgery, University Hospitals Coventry and Warwickshire National Health Service (NHS) Trust, Coventry, GBR

**Keywords:** distal end radius plating, extensor indicis tendon transfer, extensor tendon rupture, hand function, interposition graft

## Abstract

We present a case of a patient who sustained a distal radius fracture and underwent volar plate fixation. Despite initial non-operative management, subsequent corrective osteotomy was required due to malunion. Eighteen months later, the patient presented with an inability to extend the thumb, leading to a diagnosis of extensor pollicis longus (EPL) tendon rupture.

During the planned EPL tendon transfer procedure, an unexpected rupture of the extensor indicis proprius (EIP) tendon was discovered. The EIP tendon was then utilized as an interposition graft between the musculotendinous junction of the extensor indicis muscle and the EPL tendon to restore the function of EPL.

This case highlights the potential for multiple tendon ruptures following distal radius fracture treatment, even in the absence of overt clinical signs. It emphasizes the importance of thorough preoperative assessment and intraoperative vigilance to identify and address such complications.

## Introduction

Fractures of the distal radius are a common orthopedic injury [1], often requiring surgical intervention for adequate reduction and stabilization. Volar plate fixation is a widely used technique for these fractures [2]. A recognized complication of this fracture and its treatment is the subsequent rupture of the tendon of the extensor pollicis longus (EPL), which can lead to substantial morbidity [3]. If the rupture leads to functional problems for the patient, a common and reliable treatment is the transfer of the extensor indicis proprius (EIP) tendon.

While EPL tendon rupture is a well-documented complication, the coincident intraoperative finding of rupture of the EPL and EIP tendons, without prior clinical suspicion, represents a rare and underexplored phenomenon, underscoring the need for heightened intraoperative vigilance.

Here, we report a rare case of coincident rupture of both EPL and EIP tendons following volar plating for a distal radius fracture, identified at the time of the tendon transfer procedure and its operative management by using the EIP tendon as an interposition graft between EIP muscle and EPL tendon to restore the function of EPL.

## Case presentation

A 50-year-old man sustained a closed fracture of his right distal radius in 2019. Due to the coincident development of thyrotoxicosis and then the COVID lockdown, the injury was treated non-operatively and proceeded to malunion. He underwent a corrective osteotomy a year after the injury. 

**Figure 1:**
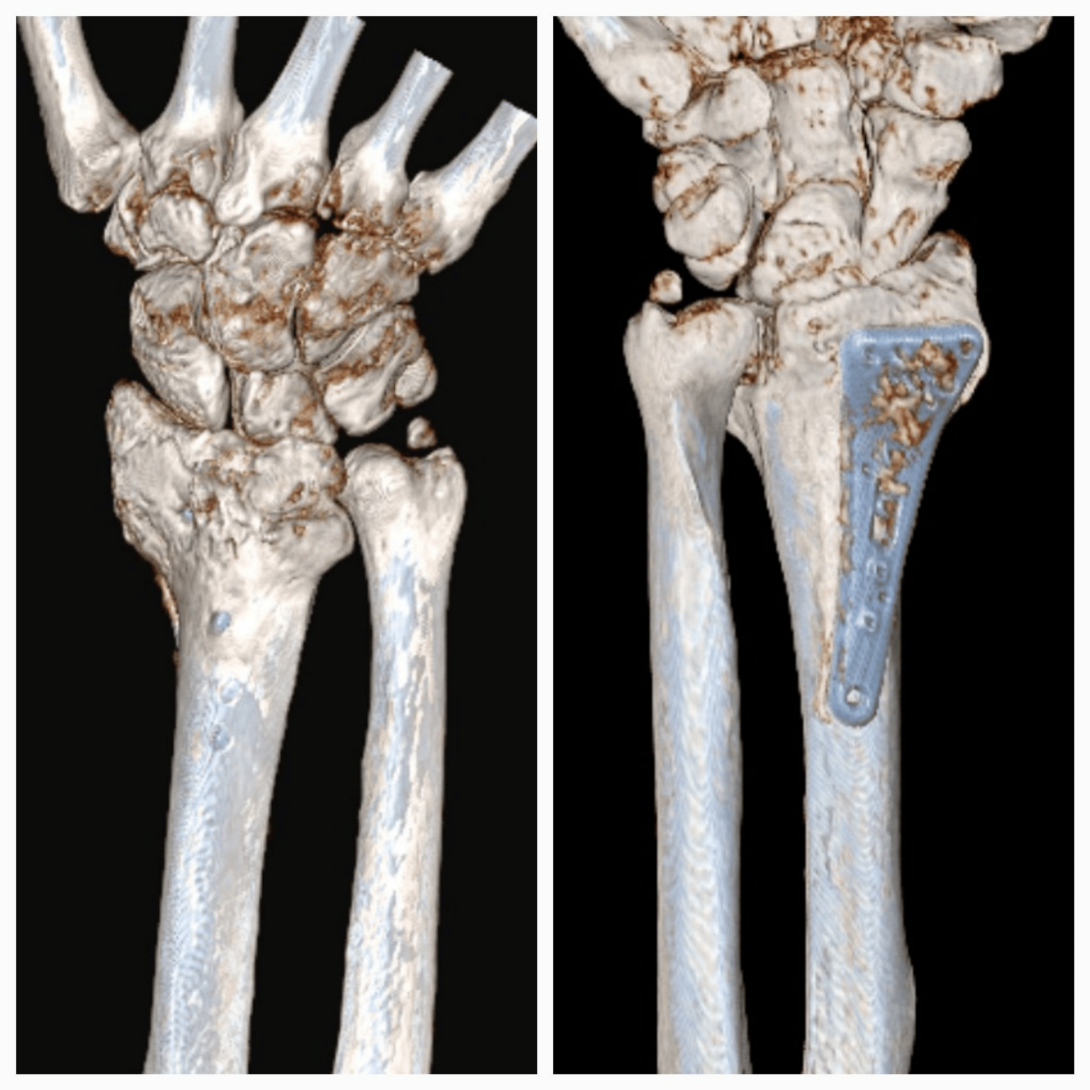
CT scan showing prominence of at least one of the screws dorsally, surrounded by callus.

Twenty-two months later, he noticed that he “could not move his thumb properly.” Examination revealed an inability to raise the thumb out of the plane of the hand, and a diagnosis of a rupture of the EPL tendon was made (Figure 1).

A plan was made for an EIP to EPL tendon transfer to improve the function of the patient’s thumb. Due to post-COVID delays, he remained on the elective waiting list for 18 months. On review on the day of surgery, he was able to extend all of his fingers and independently extend his index finger with the other fingers flexed, suggesting a functional extensor indicis. 

**Figure 2:**
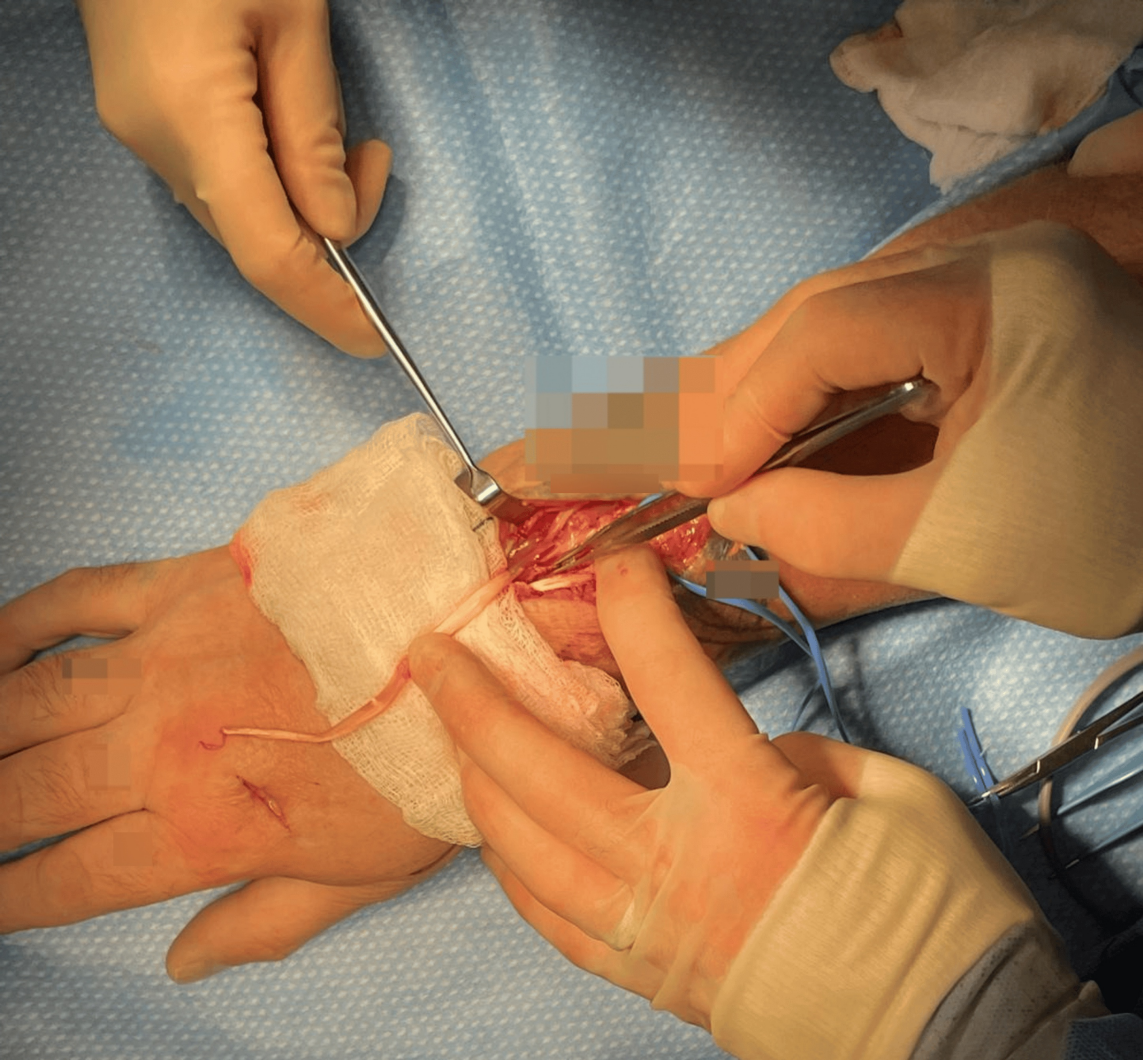
Intraoperative images: ruptured extensor indicis proprius (EIP) at musculotendinous junction with scarring interposed between.

Following the harvest of the EIP tendon via step incisions on the dorsum of the hand, the more proximal part of the tendon was explored and found to have ruptured, with scarring interposed between the tendon ends (Figure 2).

**Figure 3:**
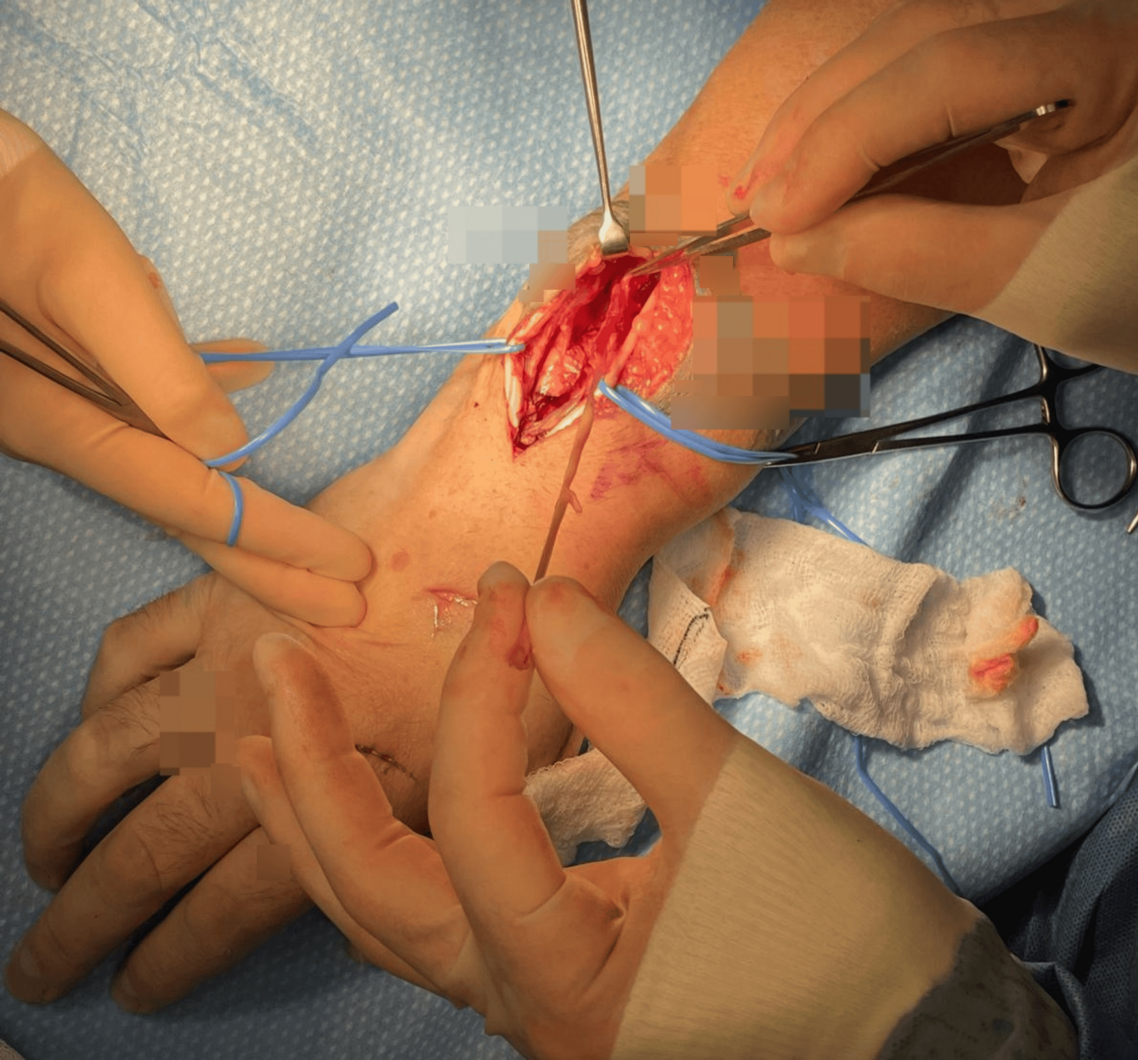
Attrition signs of other digit extensor tendons.

Furthermore, the adjacent extensors to the other digits showed signs of attrition (Figure 3).

No screws from the volar plating were palpable through the dorsal wound, only callus. 

**Figure 4:**
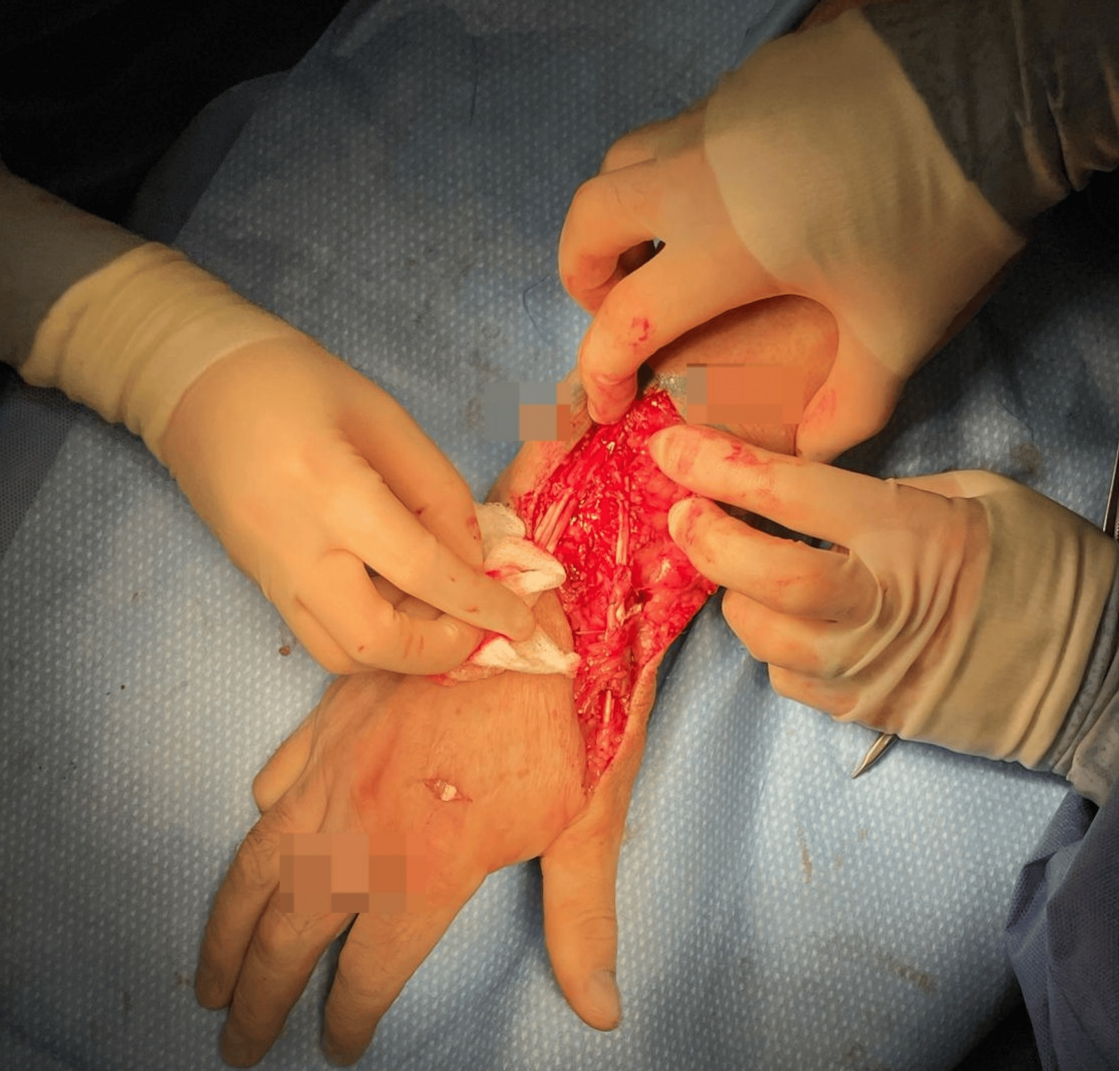
Excision of proximal scarred part of extensor indicis proprius (EIP) and interposition graft before standard Pulvertaft weave transfer of EIP to extensor pollicis longus (EPL).

Fortunately, there was sufficient tendon length in extensor indicis to allow the abnormal proximal scarred part to be resected and then reattached to itself before the transfer was completed by weaving the distal part of extensor indicis to EPL, as in a standard procedure (Figure 4).

**Figure 5:**
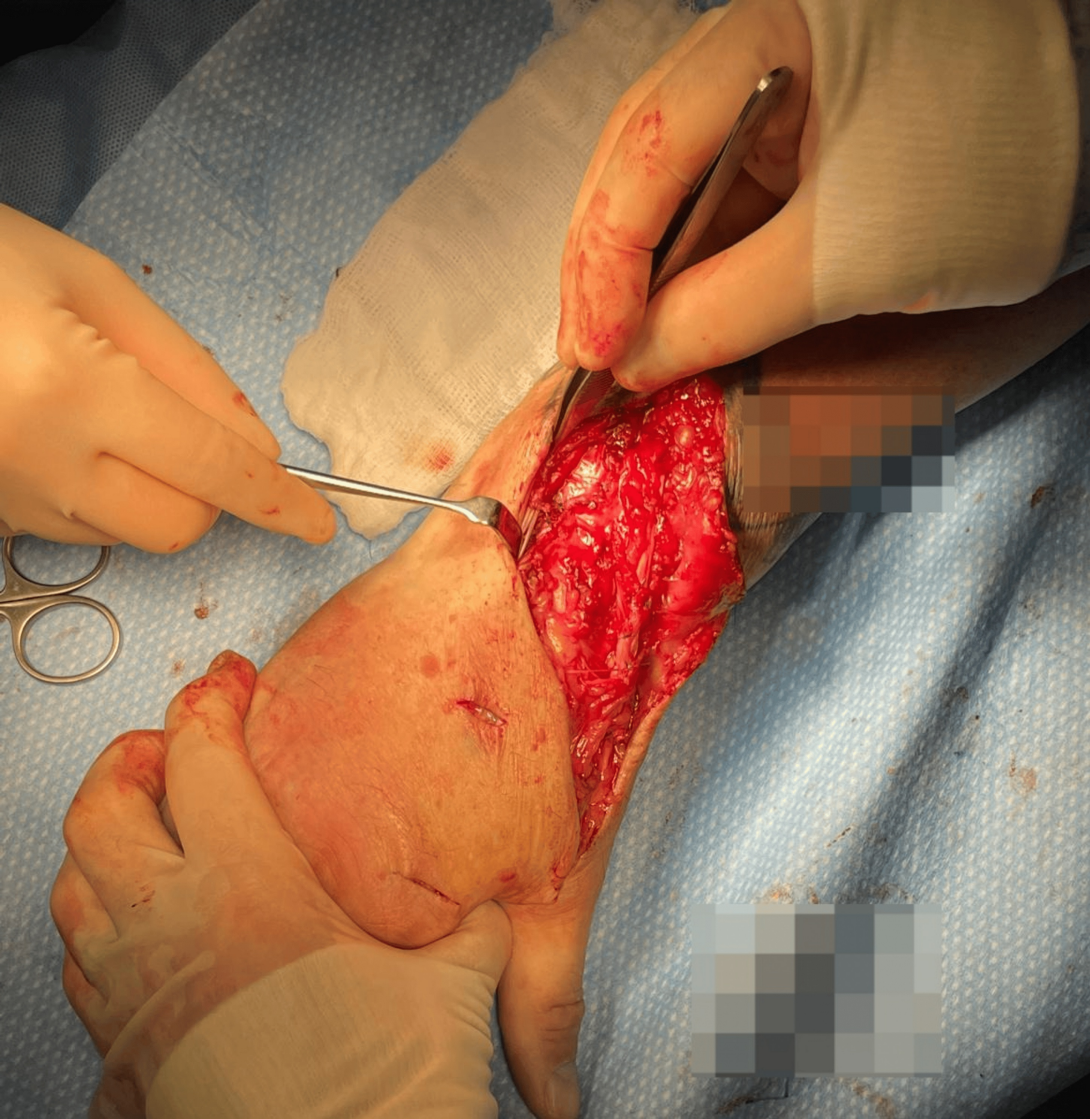
Repair of extensor retinaculum deep to extensor tendon to prevent further attrition.

The extensor retinaculum was repaired deep into the extensor tendons to try to protect them from further attrition (Figure 5).

The transfer remained intact and functional on clinic review at six weeks. 

## Discussion

This case report describes a patient who was found to have a rupture of both the EIP and EPL tendons following treatment of a distal radius fracture with volar plate fixation. Surgical management involved utilizing the EIP tendon as an interposition graft between the musculotendinous junction of the extensor indicis muscle and the EPL tendon to restore the function of EPL. 

A thorough preoperative assessment is crucial for identifying potential abnormalities and minimizing intraoperative surprises. As noted, the patient demonstrated full finger extension and independent index finger extension with other fingers flexed during the preoperative evaluation, suggesting a functional EIP. However, intraoperative findings revealed a ruptured EIP near the musculotendinous junction, unexpectedly held in functional continuity by interposed scar tissue.

Preoperative ultrasound can be an invaluable diagnostic modality in such scenarios. Notably, even preoperative ultrasound imaging can be inconclusive in rare cases due to the presence of scar tissue mimicking structural continuity.

Intraoperatively, while utilizing the EIP tendon as a graft to bridge the EPL tendon gap was considered, the significant retraction of the EPL muscle belly due to the delayed presentation rendered the graft length insufficient. Therefore, an alternative approach was employed, utilizing the EIP tendon as an interposition graft between the musculotendinous junction of the extensor indicis muscle and the proximal EPL tendon. This strategy was deemed more feasible and likely to yield better functional outcomes.

Preoperatively, the patient was informed of and consented to the potential removal of implanted hardware. However, during the procedure, no screws were palpable within the dorsal wound, only callus formation. Therefore, the implanted hardware was left undisturbed.

The rate of EPL rupture requiring tendon transfer from the EIP has been varied from study to study, ranging from 0.29% to 5.7% [3-6].

Although rare, the coincident rupture of more than one tendon has been reported on several occasions [7], for example, Concomitant Extensor Pollicis Longus and Extensor Digitorum Longus Tendon Rupture, Rupture of Both Extensor Indicis Tendons. However, the author could not find any documented incident of both EPL and EIP rupture following distal radius volar plate open reduction and internal fixation (ORIF) in the literature.

Tendon rupture following a radius fracture can occur through several mechanisms. Direct trauma from sharp bone fragments or surgical instruments can lacerate tendons, particularly the EPL tendon [8]. Indirectly, vascular compromise can lead to tendon ischemia and necrosis, while mechanical stress from narrowed tendon sheaths or prominent hardware can cause tendon irritation and rupture [9]. Additionally, muscle imbalance can place excessive strain on tendons, increasing the risk of rupture [8]. To minimize the risk, awareness of “high-risk” holes and appropriate minor alterations in surgical technique, for example, opening the third compartment during volar plate fixation depending on the prominence of the screw, repositioning the EPL from its groove, and then closing the compartment by suturing the retinaculum has been suggested in some occasion [10].

## Conclusions

This case highlights the critical importance of meticulous preoperative evaluation and vigilant intraoperative observation to detect and appropriately manage potential concomitant tendon injuries, even when pre-operative clinical findings may be misleading. While the concurrent rupture of the EPL and EIP tendons is an uncommon occurrence, surgeons must remain cognizant of such unexpected intraoperative findings. These situations necessitate a dynamic and adaptable approach to surgical planning and execution to ensure optimal patient outcomes.
